# Fellow-Workers and Their Doings

**Published:** 1914-05-30

**Authors:** 


### ROUND THE WORLD.

### Fellow-Workers and their Doings.

[The Editor Invites Early Information and Contributions Marked "Fellow-Workers."]

Dr. Dan. McKenzie, of the Central London Throat and Ear Hospital, has lately wandered into the fascinating by-paths of medical history and given special attention to the origin of the association of so many wells and springs with healing. Recently summarising the results of his inquiries in an interesting paper to the Royal Society of Medicine, Dr. McKenzie pointed out that primitive peoples have commonly connected the origin of life with water, and that it is not improbable that on this account alone wells, rivers, and springs were considered to have recuperative and life-giving principles by simple-minded folk. With regard to the offerings in the form of bits of clothing and other valueless objects commonly left by invalids at healing springs, Dr. McKenzie suggests that these are, or were originally, intended to act as a sort of permanent link between the sufferer and the source of the health-giving emanation.

Relating his observations on the use of phylacogens in rheumatic conditions in the *Practitioner* (May 1914), Dr. A. B. Flett reports hopefully of this method, and gives notes of several cases illustrating its successful application. Dr. Flett defines phylacogens as "aqueous solutions of derivatives generated by bacteria grown on artificial media"; thus, as he points out, they are neither vaccines nor sera. The rheumatism phylacogen is said to be made by "combining the metabolic substance obtained from the growth of the *Streptococcus rheumaticus* with an equal quantity of the mixed infection product." Apparently several doses of phylacogen are required under ordinary circumstances, the administration being by either subcutaneous or intravenous injection.

Dr. H. C. Malleson, M.R.C.S., L.D.S., who now takes up work at Guy's Hospital as assistant dental surgeon, has occupied the post of demonstrator of dental surgery there lately. He is also dental surgeon to Christ's Hospital, and has the distinction of having been awarded a travelling dental scholarship at "Guy's."

The new member on the staff of the Infants' Hospital, Westminster, Dr. W. M. Feldman, who has just been elected assistant physician there, is a London Hospital man, who since qualifying has taken special interest in infant care. As a medical inspector to the Whitechapel Foundling School, surgeon to the Royal Maternity Hospital, and consulting physician to the Stepney Jewish School, Dr. Feldman has had ample opportunities of gaining that experience which he has embodied in his book on "The Child: Its Rearing, Development, and Ailments." A literary and statistical study of Jewish children from his pen appeared in *The Child* of last year.

A popular Leeds practitioner, Mr. Edmund Robinson, M.R.C.S., has just retired from active work with the intention, it is understood, of travelling for a while before settling down into private life. In his practice Mr. Robinson was for some time associated with Mr. Wheelhouse, the eminent surgeon, whilst his chief public appointment was that of medical officer to the Post Office, in which connection he made important observations on the occupation neuroses of officials in the telegraph department.

Mr. Lynn Thomas, of Cardiff, has been working out a new way of performing the important operation of prostatectomy, which he has now outlined in a brief communication to the *Lancet*. Of recent years prostatectomy has been increasingly relied on to relieve the discomforts and avoid the dangerous complications of prostatic hypertrophy, and any improvements in technique which will enable the operation for the removal of the diseased gland to be carried out more safely or expeditiously will be welcomed by practitioners everywhere. Mr. Lynn Thomas is surgeon to Cardiff Infirmary and consulting surgeon to the King Edward VII. Welsh National Memorial; he is an F.R.C.S.Eng., and was made a C.B. in 1900.

The first clinical assistant to be appointed to the neurological department at St. Mary's Hospital under the new regulation is Dr. C. M. Wilson, M.D., M.R.C.P., who was formerly medical registrar there. Dr. Wilson held several resident appointments at St. Mary's Hospital, was at one time editor of the *St. Mary's Hospital Gazette*, and last year had the distinction of carrying off the gold medal at the M.D.Lond. examination.

Dr. J. Nachbar, the new medical superintendent of St. George's Hospital, has made himself very well known by his editorial and secretarial duties at the Royal Society of Medicine. He returns to the institution where he studied medicine some twenty-five years ago. Previously he was at Cambridge, where his name appeared fourteenth on the list of wranglers in 1888. Dr. Nachbar became an M.D.Cantab. in 1898.

Of the three medical practitioners who have just been appointed inspectors for the Board of Control in connection with the Mental Deficiency Act, Dr. Robert W. Branthwaite, M.D.Brux., M.R.C.S.Eng., D.P.H., is a well-known Home Office official and authority on the care of inebriates, holding the appointments of inspector and inspector of prisons under the Inebriates Act.

Dr. S. E. Gill, M.D.Lond., is assistant physician to the Nottingham General Hospital and senior inspector to the Nottingham Education Committee. Formerly he was engaged in work under the Royal Commission for the Feeble-Minded.

Mr. Cecil H. M. Hughes, who has lately been appointed assistant anæsthetist to King's College Hospital, is a member of the staff at Westminster Hospital. Mr. Hughes as anæsthetist to the Paddington Green Children's Hospital has had special experience with those of tender years. On taking up his new work he has found it necessary to give up his connection with "Westminster," where he was a student before qualifying M.B., B.S.Lond., and M.R.C.S.Eng.

Mr. H. H. Folker, M.R.C.S., crowns his active work at the North Staffordshire Infirmary by his appointment to the position of consulting ophthalmic surgeon to that institution; whilst Mr. R. H. Dickson, F.R.C.S.I., has gained his promotion to the post of ophthalmic surgeon there. At the same time Dr. S. McMurray, M.B.Irel., F.R.C.S.Edin., has been elected assistant ophthalmic surgeon to fill the vacancy thus occasioned.

